# Laser Spot Center Location Method for Chinese Spaceborne GF-7 Footprint Camera

**DOI:** 10.3390/s20082319

**Published:** 2020-04-18

**Authors:** Chaofeng Ren, Junfeng Xie, Xiaodong Zhi, Yun Yang, Shuai Yang

**Affiliations:** 1 College of Geological Engineering and Geomatics, Chang’an University, Xi’an 710054, China; rencf@chd.edu.cn (C.R.);; 2 Satellite Surveying and Mapping Application Center, NASG, Beijing 100048, China; 3 Beijing QZ Robotics Co., Ltd., Beijing 100085, China

**Keywords:** GF-7 satellite, spot center extraction, footprint camera, gaussian fitting, satellite laser altimeter

## Abstract

The Gaofen-7 (GF-7) satellite is equipped with two area array sensor footprint cameras to capture the laser altimeter spot. In order to establish a direct correspondence between the laser data and the stereo image data, a new method is proposed to fit the center of the spot using the brightness difference between the spot image and the footprint image. First, the geometric registration between the spot image and the footprint image is completed based on feature matching or template matching. Then, the brightness values between the two images are extracted from the corresponding image position to form a measurement, and the least squares adjustment method is used to calculate the parameters of the brightness conversion model between the spot image and the footprint image. Finally, according to the registration relationship, the center of the identified spots is respectively positioned in the footprint images, so that the laser spots are accurately identified in the along-track stereo footprint images. The experimental results show that the spot error of this method is less than 0.7 pixel, which has higher reliability and stability, and can be used for a GF-7 satellite footprint camera.

## 1. Introduction

The geometrical positioning accuracy of surveying and mapping satellites is mainly limited by the attitude performance of the satellite platform which affects the precision of orientation parameters [1]. Due to the base-to-height ratio, platform stability, and other factors, the vertical accuracy of optical stereoscopic mapping is difficult to meet application requirements [2]. As a high-precision ranging instrument, laser altimeters can obtain high-precision vertical measurement information, and have been widely used in the field of aerospace photogrammetry [3]; these include the Geoscience Laser Altimetry System (GLAS) system [4], the Mars Orbiter Laser Altimeter (MOLA) system [5], China’s ZY3-02 satellite system [6], and Gaofen-7 (GF-7) satellite system. Xie et al. [7] verified that the laser precision of the ZY3-02 satellite system is about 2~3 m in areas with a slope less than 2°, and the absolute accuracy is better than 1 m in flat areas after calibration. Consequently, integrating the stereo imagery and laser altimeter data has the potential to generate better geometrical positioning accuracy [3], especially for areas where it is difficult to obtain ground control points. However, due to various errors, such as attitude errors, synchronization errors, calibration errors, and environmental errors, the calculated laser ground points may be inconsistent with the real locations. Thus, non-conjugated points are generated by back-projecting the laser ground points to the stereo images [3]. These deviations have caused great difficulties in constructing a combined adjustment model of laser data and stereo image data.

GF-7 satellite is China’s first sub-meter resolution optical stereo mapping satellite of China’s high-resolution earth observation system. It was successfully launched at 11:22 on 3 November 2019. The satellite’s payload equipment includes two linear charged-couple device (CCD) cameras to obtain forward-view and backward-view stereo images, one dual-beam laser altimeter, four-band multispectral cameras, and two area array sensor footprint cameras to capture the altimeter spot. GF-7 satellite establishes a direct correspondence between the laser data and the stereo image data through the footprint camera. This paper studies how to locate the center of the laser spot and establishes a geometric conversion model between the center of the spot and the footprint image.

Previous research involving laser spot center location relied on methods that used the edge information of the spot image to fit the spot center, such as the gravity method [8], circle fitting method [9,10,11], parabolic fitting method [12], Gaussian fitting method [13], and three-dimensional arctangent function fitting method [14]. The fitting accuracy of the gravity center method and the circle method is greatly affected by the edge of the spot image [15]. The parabolic fitting method and the Gaussian fitting method are more suitable for spot images with small-size and Gaussian-distributed spot images [16]. In contrast, the three-dimensional arctangent function fitting method is suitable for a large-size spot image and non-Gaussian-distributed spot images. The above methods can accurately fit the spot center, depending on whether the spot image can be accurately segmented from its background image. However, after long-distance transmission from the satellite to ground, when laser pulses are captured by the footprint camera, a significant scattering phenomenon occurs at the edge of the spot image, making it difficult to distinguish the spot image from the background image. In actual satellite data processing, it is difficult to achieve ideal segmentation results using tradition region segmentation methods, such as fixed threshold method, adaptive threshold method [17], and GrabCut method [18], which make the partially fitted spot centers deviate from the real position, and thus the accuracy requirements cannot be met.

As stated above, developing a new method to locate the laser spot center of the GF-7 footprint image is important. Therefore, based on the analysis of the working mode of the GF-7 footprint camera, a brightness conversion model between the spot image and the footprint image was established. After that, we propose a method that first completes the geometric registration between the spot image and the footprint image, then combines the brightness difference of the corresponding pixels to adaptively calculate the conversion model parameters and obtain the spot center position based on the results. The method does not need to segment the spot image from the background, and avoids fitting errors caused by edge segmentation errors. Experiments demonstrate that the method has better accuracy than previous methods. It can be used to automatically identify and locate the spot center of the GF-7 footprint image. 

## 2. Materials and Methods 

### 2.1. Specifications of GF-7 Footprint Camera

**Figure 1:**
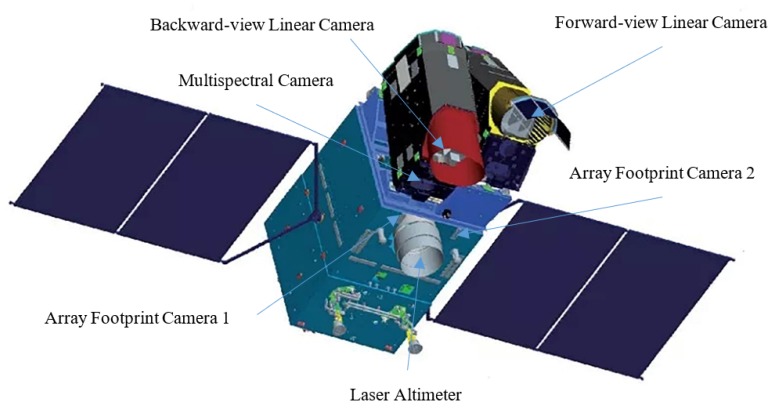
Schematic diagram of the installation of the laser altimeter and the footprint camera onboard the Gaofen-7 (GF-7) satellite.

GF-7 is the first satellite equipped with a footprint camera to capture the spot of the laser altimeter in China. After identifying and locating the center of the spot image, the texture information of the spot image can be matched with the linear stereo images, combining the laser altimeter data to form a high-precision global ground control point, which is very useful for improving the geo-positioning accuracy of Earth topographic models. A schematic diagram showing the installation of the laser altimeter and the footprint camera of GF-7 is illustrated in Figure 1, wherein the pointing of the laser altimeter is substantially parallel to the visual axis of the footprint camera.

**Figure 2:**
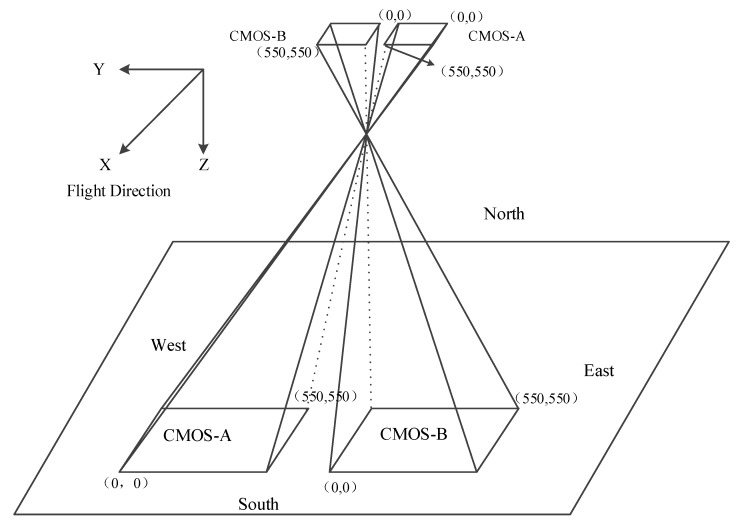
Schematic diagram of the footprint camera onboard the GF-7 satellite.

As illustrated in Figure 2, the two footprint cameras are horizontally arranged in the flight direction of the GF-7 satellite orbit, which are respectively used to capture laser spots on both sides of the orbit. The imaging device of the footprint camera is an area array Complementary Metal Oxide Semiconductor (CMOS), and the image size obtained by the footprint camera is 550 × 550 pixels. The pixel size of the footprint camera is 16.5 μm, and the corresponding ground sampling distance (GSD) is 3.2 m, which means the ground distance corresponds to 1 pixel of the footprint camera.

**Figure 3:**
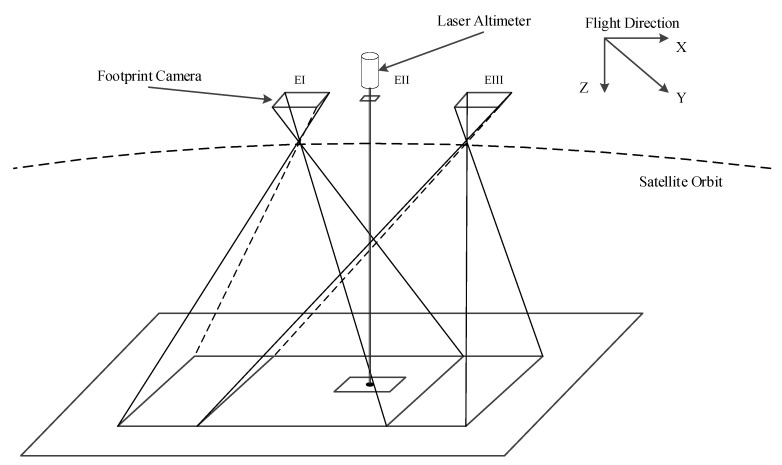
Schematic diagram of the working mode of the footprint camera and laser altimeter.

The footprint camera of GF-7 uses three adjacent exposure modes, EI, EII, and EIII, in a single exposure circle, as shown in Figure 3. EI and EIII are full-frame imaging exposure modes, and the obtained footprint image size is 550 × 550 pixels. In the same exposure circle, EI and EIII have about a 90% overlap area, forming an along-track stereo image. Different from EI and EIII, EII is a partial imaging exposure mode, and the exposure time is synchronized with the laser altimeter pulse time. EII is specifically designed to capture the laser spot image, and its image size is 84 × 84 pixels. 

**Figure 4:**
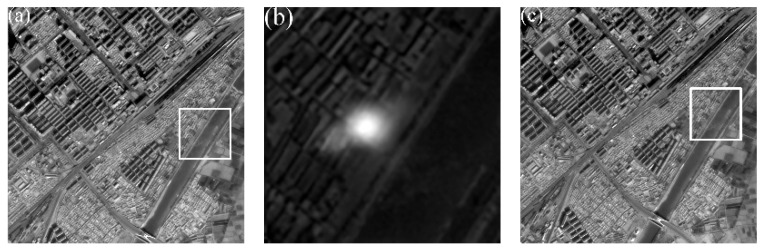
Three adjacent exposure footprint images. (**a**) footprint image EI; (**b**) spot image EII; (**c**) footprint image EIII.

Figure 3 is a schematic diagram of one of two footprint cameras. Figure 4 shows the final obtained footprint image and laser spot image after an exposure cycle is completed. The white rectangles in Figure 4a,c are the coverage of the spot in Figure 4b. The white spot in Figure 4b is the laser pulse information captured by the footprint camera.

### 2.2. Laser Spot Center Location Method of GF-7 Satellite

#### 2.2.1. Geometric Registration Model

The geometric registration model from the spot image to the footprint image determines the corresponding position of the spot center on the footprint image. The projective mapping between the two images or planes is known as the homograph matrix [19,20]. The conversion from the image coordinates of the spot image to the image coordinates of the footprint image can be expressed as Equation (1).

(1) $$\left[\begin{array}{c} u_{f}\\ v_{f}\\ 1 \end{array}\right]=H\left[\begin{array}{c} u_{s}\\ v_{s}\\ 1 \end{array}\right]$$

where ${\left[\begin{array}{cc} u_{s} & v_{s} \end{array}\right]}^{T}$ are the image coordinates of a pixel in the spot image, and ${\left[\begin{array}{cc} u_{f} & v_{f} \end{array}\right]}^{T}$ are the corresponding image coordinates of the footprint image; the detailed definition of the homography matrix is $H=\left[\begin{array}{ccc} h_{11} & h_{12} & h_{13}\\ h_{21} & h_{22} & h_{23}\\ h_{31} & h_{32} & 1 \end{array}\right]$.

The image matching of the spot image and footprint image adopts the scale-invariant feature transform (SIFT) feature [21,22]. Siddique et al. [23] proposed an automatic registration method for synthetic-aperture radar (SAR) images and optical images and achieved good results. In this paper, both the spot image and footprint image are optical images, so the traditional Random Sample Consensus (RANSAC) algorithm [24] is used to remove erroneous matching points. When the number of matching points is sufficient (at least 4), the parameters of matrix ‘H’ can be estimated by RANSAC-based homography [25]. However, the spot image is small and often obscured by shadows or lack of texture; it is not guaranteed that each image pair can obtain a sufficient number of matching points. Therefore, when the image matching based on the SIFT feature fails, the template matching method is used as a subsequent solution. Then, the geometric transformation from the spot image to the footprint image is only a two-dimensional translation, and the definition of the homography matrix is $H=\left[\begin{array}{ccc} h_{11} & 0 & 0\\ 0 & h_{22} & 0\\ 0 & 0 & 1 \end{array}\right]$.

Given the large brightness difference between the spot image and the footprint image, the reliability of template matching on the original image is too low. Thus, a gradient image is introduced instead of the original image for template matching. This paper uses the normalized correlation coefficient (NCC) method [26,27] as the similarity measure for template matching, since the method can better resist the change in brightness, as shown in Equation (2).

(2) $$r(u,v)=\frac{\sum_{i=0}^{w-1}\sum_{j=0}^{h-1}\left[f\left(i,j\right)-{\bar{f}}_{u,v}\right]\left[t\left(i+u,j+v\right)-\bar{t}\right]}{\sqrt{\sum_{i=0}^{w-1}\sum_{j=0}^{h-1}{\left[f\left(i,j\right)-{\bar{f}}_{u,v}\right]}^{2}{\left[t\left(i+u,j+v\right)-\bar{t}\right]}^{2}}}$$

where t is the template image, which is the gradient spot image; f is the search region, which is within the gradient footprint image; $\bar{t}$ is the mean value of the template image and ${\bar{f}}_{u,v}$ is the mean value of f(i,j) in the search region under the template image; r(u,v) is the correlation coefficient at point (u,v) for f and t.

The spot image EII is used as a template image, and the NCC is calculated pixel by pixel in the region of the footprint image EI and EIII. The image coordinate corresponding to the maximum NCC value is the translation from the spot image to the footprint image, as shown in Equation (3).

(3) $$\left(u,v\right)=\left(u,v\right)|{\mathrm{NCC}}_{\max}$$

#### 2.2.2. Brightness Conversion Model

Since the pointing direction of the laser altimeter is perpendicular to the ground, the laser spot finally captured by the footprint camera has a circular distribution and edge attenuation. This study defines the spot image itself as a two-dimensional Gaussian distribution, as shown in Equation (4).

(4) $$I_{s}\left(x,y\right)=K\cdot \exp\left\{-\left[\frac{{\left(x-x_{0}\right)}^{2}+{\left(y-y_{0}\right)}^{2}}{\sigma^{2}}\right]\right\}$$

where $I_{s}(x,y)$ is the brightness value at the spot image (x,y); K is the magnitude of the intensity of the Gaussian distribution; σ is the standard deviation of the Gaussian kernel function.

Equation (4) shows the brightness distribution of the laser on the spot image, and the image coordinates $\left(x_{0},y_{0}\right)$ are the theoretical center positions of the spot image. Affected by factors such as footprint camera parameter settings, solar illumination, and ground environment, there are obvious brightness differences between the spot image and the footprint image. If the influence of the laser spot is not considered, the brightness conversion model between the spot image and the footprint image can be defined as a one-dimensional linear transform [28]. In addition, based on the principle of image superimposition, the spot image acquired by the footprint camera can be considered to be formed by the laser spot information and the surrounding environment of the ground. Therefore, after removing the laser effect, the brightness conversion model between the spot image and the footprint image can be defined as Equation (5).

(5) $$I_{3}(x,y)=\left[I_{2}\left(x,y\right)-I_{s}(x,y)\right]g+o$$

where $I_{2}(x,y)$ is the brightness value at the spot image (x,y); $I_{3}(x,y)$ is the brightness value of the footprint image corresponding to the spot image coordinates (x,y); (g,o) are the gain and offset of the brightness conversion model from image $I_{2}$ to $I_{3}$.

$I_{s}(x,y)$ in Equation (5) is replaced with the detailed definition of $I_{s}(x,y)$ in Equation (4). The brightness difference from the spot image to the footprint image can be defined as Equation (6).

(6) $$\Phi \left(x_{0},y_{0},\sigma ,K,g,b\right)=\left\{I_{2}-K\cdot \exp\left\{-\left[\frac{{\left(x-x_{0}\right)}^{2}+{\left(y-y_{0}\right)}^{2}}{\sigma^{2}}\right]\right\}\right\}g+o-I_{3}$$

Equation (6) is the brightness difference function from the spot image EII to the footprint image EIII, and the same is true for EII to EI.

**Figure 5:**
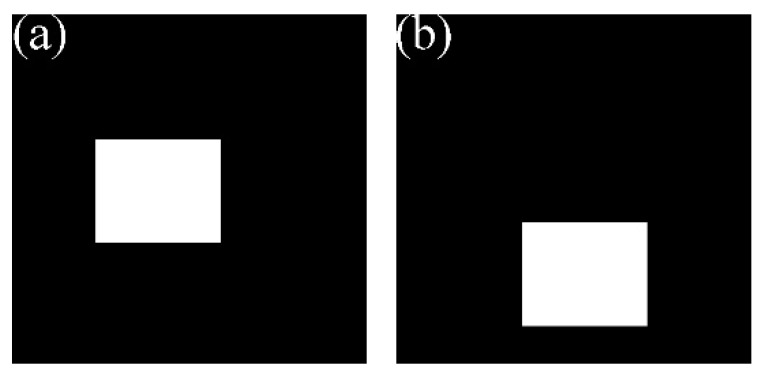
Spot area and non-spot area of footprint image. (**a**) Spot area of footprint camera 1; (**b**) spot area of footprint camera 2.

After the geometric registration of the spot image and the footprint image is completed, the brightness values of them are extracted pixel by pixel within the coverage area of the spot image, which can be used as an observation of Equation (6). In order to ensure the robustness of the parameters estimation results in Equation (6), a quadratic adjustment method is proposed. First, by the extracted observations, the system variable (g,o) between the spot image and the footprint image is estimated by least squares adjustment. Second, the estimated results (g,o) are considered as the true values, and the parameters $\left(x_{0},y_{0},\sigma ,K\right)$ of the Gaussian distribution are estimated. Although the angle between the laser altimeter and the optical axis of the footprint camera is constant, the position of the laser spot in the spot image is basically constant, but slightly changed due to factors such as orbit attitude errors and time synchronizations errors. Therefore, the spot image is divided into a spot area and a non-spot area, as shown in Figure 5.

Figure 5a shows the spot area mask of the footprint camera 1, and Figure 5b shows the spot area mask of the footprint camera 2. For the first adjustment, the observations without laser information are used, which are extracted from the non-spot area, as shown in Figure 5a outside the white rectangle. Then, the value of $I_{s}\left(x,y\right)$ in Equation (5) is 0, and the system variable (g,o) is linearly related to the observations; it can be adjusted by the least square adjustment. For the second adjustment, the observations are extracted from the spot area, as shown in Figure 5a in the white rectangle. Since the parameters $\left(x_{0},y_{0},\sigma ,K\right)$ of the Gaussian distribution in Equation (6) have a non-linear relationship with the observations, it can be estimated by a non-linear least squares method. In the adjustment optimization, the constraints of the model parameters are shown in Equation (7).

(7) $${\left(x_{0},y_{0},\sigma_{2},K,g,b\right)}^{*}=\underset{0\leq x_{0}\leq w,0\leq y_{0}\leq h,H>0,\sigma_{2}>0}{\arg\;\min}\Phi (x_{0},y_{0},\sigma_{2},K,g,b)$$

where $\sigma_{2}$ is $\sigma^{2}$ in Equation (6); the initial value of $\left(x_{0},y_{0}\right)$ is set to the geometric center of the spot image; and the initial value of $\sigma_{2}$ and K are both set to 1.

### 2.3. Introduction to the Experimental Data

**Figure 6:**
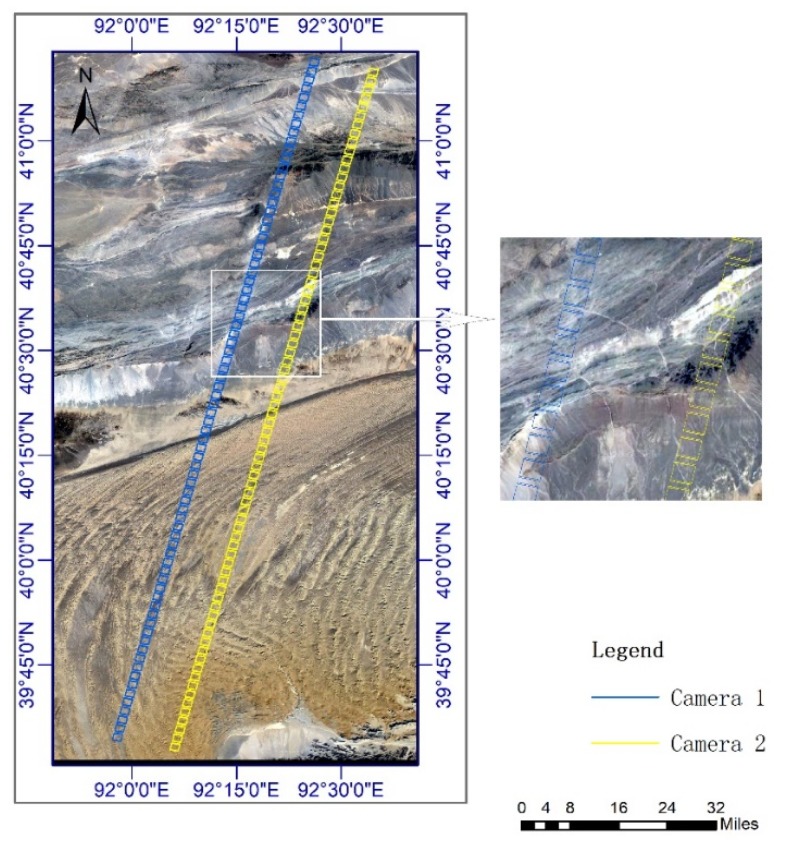
Orbit 221 experimental data.

To validate the proposed method, GF-7 spot images and footprint images of orbit 221 and orbit 295 covering the XinJiang areas in China were collected. A total of 95 effective three-exposure data was collected in orbit 221; the acquisition date was 17 November 2019. A total of 84 effective three-exposure data was collected in orbit 295; the acquisition date was 22 November 2019. For the orbit 295 dataset, as shown in Figure 6, there are two cameras footprints, which are footprint camera 1 and footprint camera 2. In a three-exposure cycle, the footprint image EI and the footprint image EIII have overlapping areas, but there are no overlapping areas in adjacent exposure cycles. 

The proposed algorithm is developed in Visual Studio 2015 C++, and the non-linear optimization uses the open source library Ceres-Solver [29] to complete the adjustment.

## 3. Results and Discussion

### 3.1. Evaluation Metric

The real spot center position in the spot image is not available, so the accuracy of the spot center extraction algorithm cannot be directly evaluated. However, in an exposure circle, footprint image EI and EIII form an along-track stereo image, so the center of the spot in footprint EI and the center of the spot in footprint EIII satisfy the epipolar constraint. This constraint arises from the fact that the pair of spot centers in footprints and the optical centers of the two footprint cameras must lie on a plane, as shown in Equation (8).

(8) $$x_{3}^{T}Fx_{1}=0$$

where $x_{3}$ is the image coordinate of the spot center in footprint image EIII; $x_{1}$ is the image coordinate of the spot center in footprint image EI; F is the fundamental matrix between EI and EIII, which can be solved by the corresponding points of the EI and EIII.

The intersection of the epipolar plane with the footprint image plane is the epipolar line, as shown in Equation (9).

(9) $$\{\begin{array}{c} l^{\prime }=Fx_{1}\\ l=F^{T}x_{3} \end{array}$$

where $l^{\prime }$ is the epipolar line corresponding to $x_{1}$; l is the epipolar line corresponding to $x_{3}$.

After laser spot location, place the image coordinates $x_{1}$ and $x_{3}$ of the spot center into Equation (9), and the distances from the laser center to the epipolar line are calculated. The average of the two distances is used as an accuracy metric for the positioning of the laser spot center.

### 3.2. Validation of the Accuracy of Geometric Registration 

**Figure 7:**
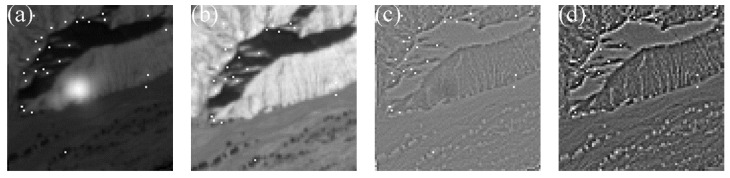
Feature matching and template matching results. (**a**) Spot image features; (**b**) footprint image features; (**c**) spot image gradient; (**d**) footprint image gradient.

On the basis of the analysis in Section 2.2.1, there are two methods, feature-based matching and template-based matching, to complete the geometric registration of spot images and footprint images, and the latter method is used only when the former method cannot obtain a sufficient number of corresponding points. In order to evaluate the geometric accuracy of the two methods, a set of three-exposure images of orbit 221 was selected for feature-based and template-based matching, and the results are shown in Figure 7.

**Figure 8:**
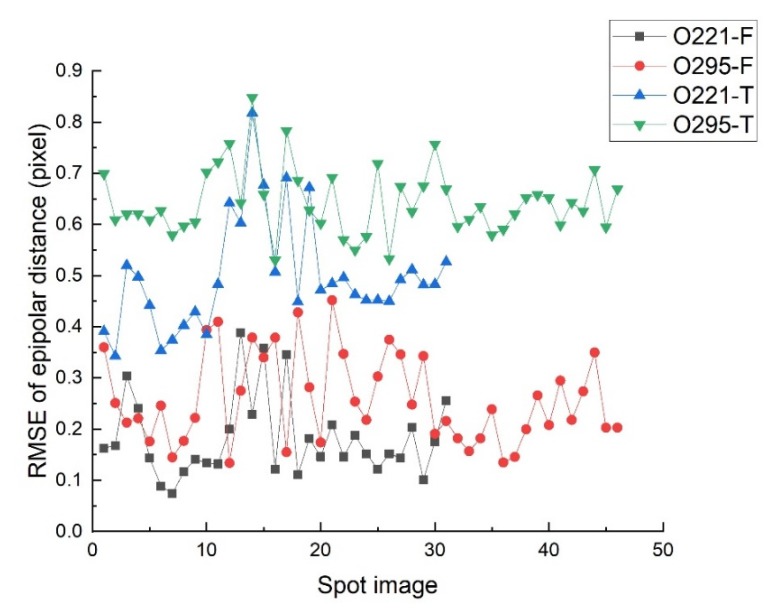
The root mean square error (RMSE) of geometric registration between spot image and footprint image.

Figure 7a,b is the matching results of the spot image and the footprint image, respectively, and a total of 29 corresponding points were obtained, as shown by the small white dots. By bringing the image coordinates of corresponding points into Equation (9), the root mean square error (RMSE) of the epipolar error is 0.073 pixel. The results show that the projection mapping relationship between the spot image and the footprint image based on the homography matrix can achieve higher accuracy. Figure 7c,d is gradient images of the spot image and the footprint image, and the white dots are corresponding points based on feature matching. In order to evaluate the geometric registration accuracy of template matching by equal condition, the translation value based on template matching is added to the image coordinates obtained by feature matching, and the RMSE of the epipolar error is 0.374 pixel. Obviously, the geometric accuracy of the template matching method is lower than the feature matching method. The reason is that the template matching simplifies the projection mapping between the spot image and the footprint image to a translation transformation, which cannot accurately simulate the local changes of the images. Using the same method as in Figure 7, the geometric registration of the remaining images of orbit 221 and orbit 295 is completed, and the RMSE of the epipolar error is shown in Figure 8.

In Figure 8, O221_F is the RMSE of the epipolar error based on feature matching of orbit 221, and O221_T is the RMSE of the epipolar error based on template matching of orbit 221. O295_F and O295_T have the same meaning. Analysis of the epipolar error curve in Figure 8 shows that the registration accuracy based on feature matching of the two orbit data is within a similar variation range, and both are significantly better than the results based on template matching. The detailed statistical results are shown in Table 1.

The results in Table 1 show that the average error of geometric registration based on feature matching is better than 0.3 pixel, and the average error of template matching is better than 0.7 pixel. The accuracy of both methods is less than 1.0 pixel.

### 3.3. Validation of the Positioning Accuracy of the Laser Spot Center

**Figure 9:**
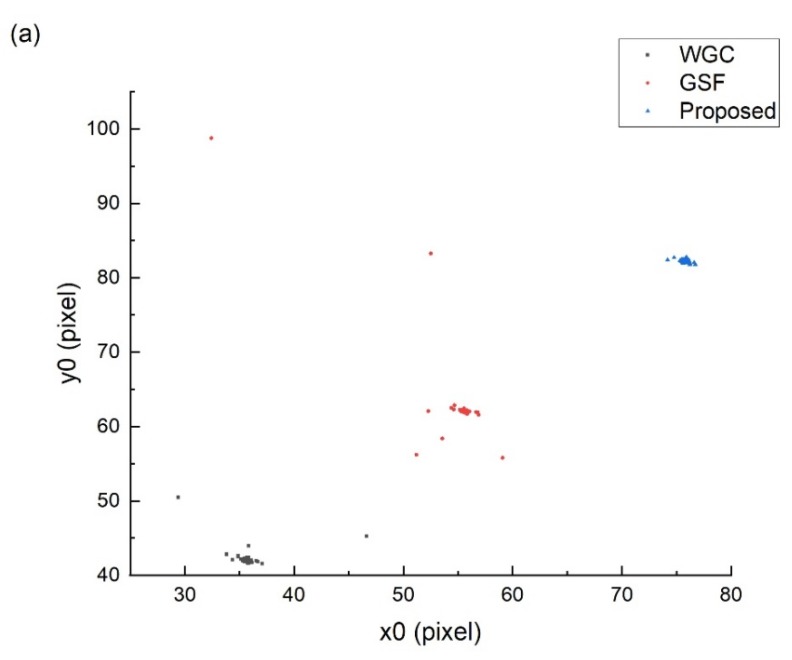

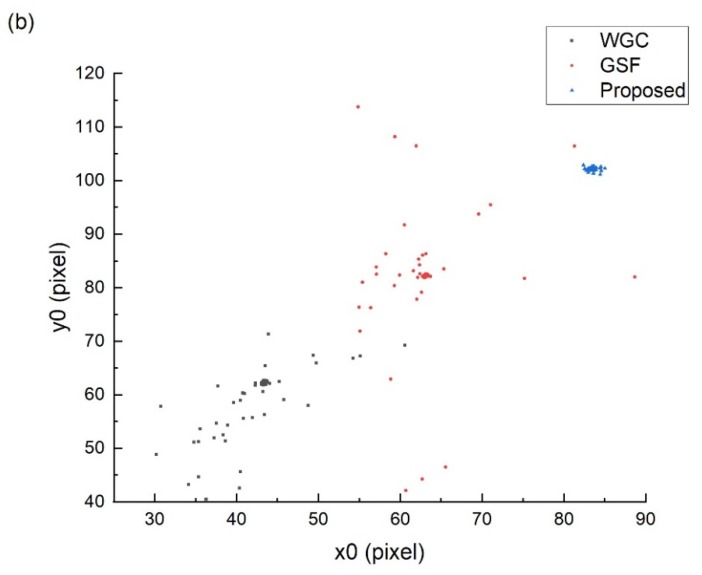
Distribution of spot center. (**a**) Orbit 221 results; (**b**) orbit 295 results.

After the geometrical registration of the spot image and the footprint image is completed, the location of the spot center is completed according to the method in Section 2.2.2. In addition, in order to compare with the proposed method in this paper, the adaptive method [17] is used to separate the spot area, and then the weighted gravity center (WGC) method [8] and the Gaussian surface fitting (GSF) method [12] are used to identify the center of the spot on the orbit 221 and orbit 295 data. The final spot center distribution is shown in Figure 9.

**Figure 10:**
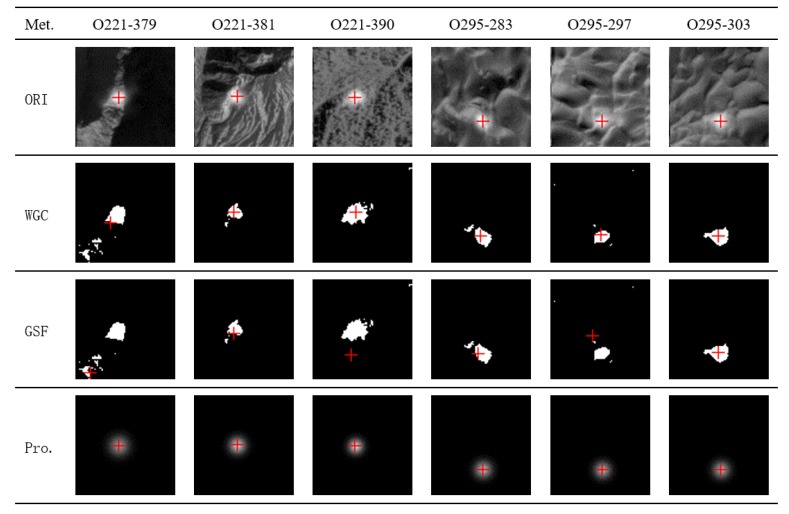
Comparison of spot center fitting results. ORI: original spot image; WGC: weighted gravity center; GSF: Gaussian surface fitting; Pro.: Gaussian distribution image by proposed method.

In order to facilitate the analysis, in Figure 9, the coordinates of the spot center identified by the GSF method and the proposed method are added with a translation value of 20 pixels and 40 pixels, respectively. Figure 9a shows the spot center distribution of footprint camera 1 obtained from the orbit 221 data, and Figure 9b shows the results of footprint camera 2 obtained from the orbit 295 data. The results show that the spot center distribution fitted by the proposed method has the best consistency, which is consistent with the theoretical design of the footprint camera, while the WCG and GSF method have obvious fitting errors. Three sets of fitting error examples were selected from orbit 221 and orbit 295 data for comparison and analysis, and the results are shown in Figure 10.

In Figure 10, the first row is the superimposed image of the original spot image and the fitting result of the spot center by the proposed method. The second row and the third row are superimposed images of the spot area separated by the adaptive method and the fitting results of the WGC and GSF methods, respectively. The fourth row is the Gaussian distribution image obtained by the proposed method. From the analysis of the second and third rows in Figure 10, it is apparent that the traditional WCG and GSF method results are very susceptible to irregular spot edges, and the spot edges obtained based on the adaptive threshold are difficult to effectively segment from the spot background, making the fitting result of the spot center obviously wrong. However, the method in this paper avoids segmenting the spot area from the spot background, and then obtains stable fitting results.

**Figure 11:**
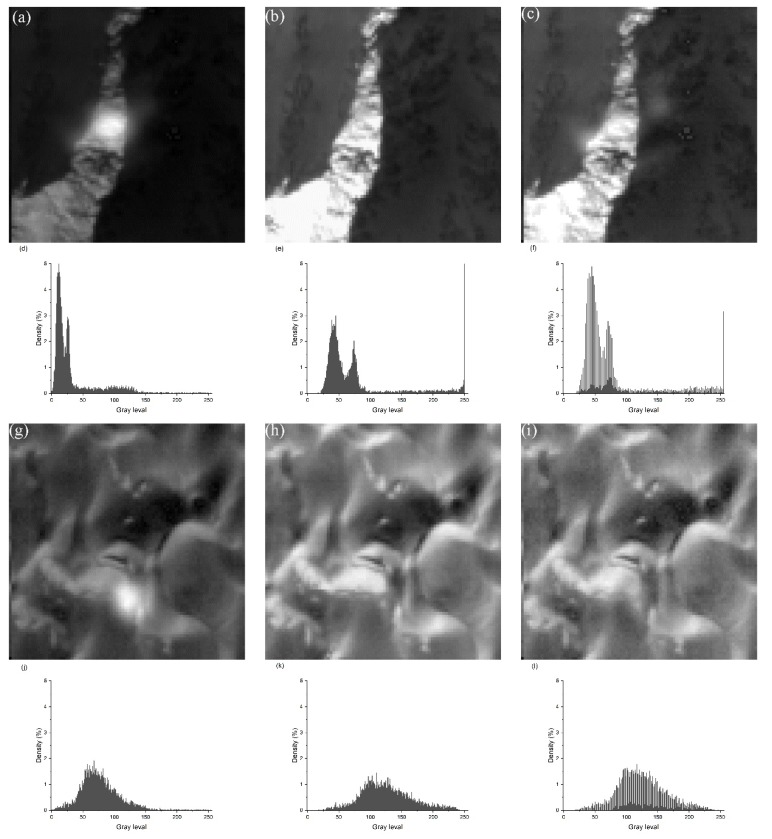
Comparison of brightness values and histograms before and after brightness conversion. (**a**) Original spot image of O221-375; (**b**) footprint image of O221-375; (**c**) spot image after conversion of O221-375; (**d**) histogram of (a); (**e**) histogram of (b); (**f**) histogram of (c); (**g**) original spot image of O295-283; (**h**) footprint image of O295-283; (**i**) spot image after conversion of O295-283; (**j**) histogram of (g); (**k**) histogram of (h); (**l**) histogram of (i).

In order to evaluate the brightness value after brightness conversion, we generated the conversion images and histograms of the “O221-379” and “O295-238” spot images in Figure 10, and the results are shown in Figure 11.

Comparing Figure 11a,b, we see that there is a significant difference in brightness between the original spot image and the corresponding footprint image. Figure 11c is a spot image after the brightness conversion, which has eliminated the brightness difference from Figure 11b. Compared with the spot image histogram in Figure 11d before conversion and the spot image histogram in Figure 11f after conversion, the same conclusion can be obtained. Thus the brightness conversion model in this paper is necessary and effective.

**Figure 12:**
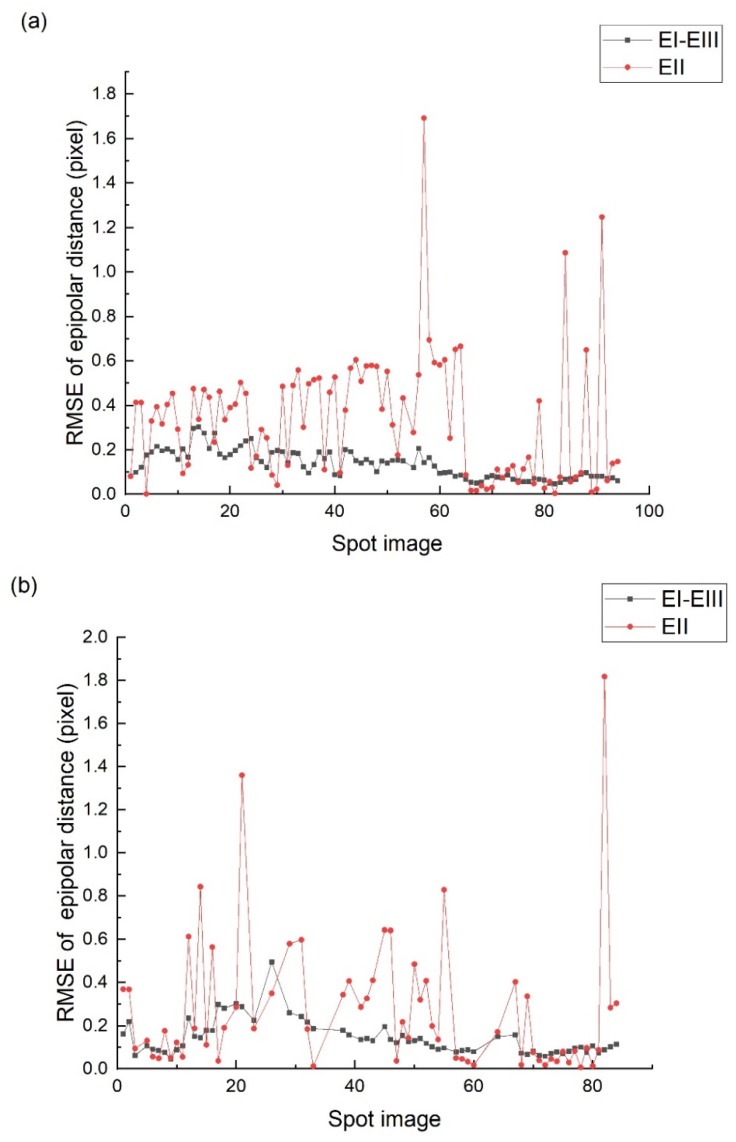
The RMSE of epipolar error of the spot center. (**a**) Orbit 221 results; (**b**) orbit 295 results.

In Figure 12, EI-EIII represents the epipolar RMSE of the corresponding points between the footprint images EI and EIII. EII represents the epipolar error of the spot center located on footprint image EI and EIII. By analyzing the data in Figure 12, it is apparent that the positioning errors of the spot center by the proposed method can be summarized into three types:

(1) The first type is that the epipolar error is consistent with the RMSE of image EI and EIII, which basically does not exceed 0.3 pixel. By analyzing the specific processing process, it is found that the results of this type are all based on the feature matching to complete the geometric registration. Therefore, after positioning the fitted spot center to the footprint image, its epipolar error is consistent with the RMSE of footprint image EI and EIII.
(2) The second type is that the epipolar error exceeds the RMSE of image EI and EIII but does not exceed 0.7 pixel. The results of this type are based on template matching to complete the geometric registration, and the accuracy of the spot center positioning is consistent with the template accuracy.
(3) The third type is the epipolar error exceeds 1 pixel. In Figure 12, a part of the spot images with an epipolar distance error more than 2 pixels is deleted for the convenience display. The epipolar error of this type is too large because its geometric registration process fails. The reason for geometric registration failure is that the original image is covered by clouds, shadows, or the image lacks texture information.

Since the method in this paper was to complete the registration and fitting between image pairs EII-EI and EII-EIII independently, the spot centers of the two image pairs are independent and uncorrelated, so that the epipolar error of the spot center can represent the actual accuracy of the spot center. However, the traditional methods such as WCG and GSF only rely on the spot image to identify the spot center, and the results are not independent. Therefore, if the fitting of the spot center fails, then the spot image and the footprint image are successfully registered. However, the spot center located on the footprint image still satisfies the epipolar constraint, and the position of the spot center cannot be evaluated by the epipolar error.

## 4. Conclusions

In this study, a new method was proposed to locate the laser spot center of the GF-7 satellite footprint camera. On the premise that the spot image and the footprint image registered, this method makes full use of the brightness difference between the spot image and the footprint image to fit the parameters of the brightness conversion model and obtains the optimal spot center. Through the analysis and verification of the experimental data, the following conclusions can be summarized:

(1) The fitting accuracy of the spot center is mainly affected by the geometric registration accuracy. The epipolar error of the spot center based on the feature matching method is less than 0.3 pixel. The epipolar error of the spot center based on the template matching method is less than 0.7 pixel.
(2) The results of this method are more reliable. The epipolar error can effectively represent the accuracy of the spot center, and the results with epipolar errors exceeding 1 pixel will be eliminated and cannot be used for the combined adjustment of laser altimeter and stereo images.

Finally, the validity and robustness of the method in this study are verified by the GF-7 satellite data, which prove that the method can be applied to the laser spot center location of GF-7 satellite footprint camera.

## Figures and Tables

**Table 1:** Statistics of geometric registration between spot image and footprint image.

Method	Max Error (pixel)	Min Error (pixel)	Average Error (pixel)
O221-F	0.389	0.073	0.182
O221-T	0.818	0.343	0.495
O295-F	0.452	0.134	0.259
O295-T	0.848	0.530	0.643
